# The Nature, Taxonomy, and Contingencies of Intimate Relationship Problems

**DOI:** 10.1007/s12110-025-09489-7

**Published:** 2025-03-24

**Authors:** Menelaos Apostolou, Loizos Katsaris, Antonios Kagialis, Loukia Constantinidou

**Affiliations:** 1 Department of Social Sciences, University of Nicosia, 46 Makedonitissas Ave, Nicosia, 1700 Cyprus; 2 Department of Psychiatry, School of Medicine, University of Crete, Heraklion, Crete Greece

**Keywords:** Relationship problems, Nature of relationship problems, Intimate relationships, Mating

## Abstract

Intimate relationships are frequently characterized by problems, which the current research aimed to identify. We first proposed an evolutionary theoretical framework to understand the nature of intimate relationship problems. Subsequently, we employed a mixed-methods approach to identify these problems. In particular, Study 1 used a combination of qualitative research methods on a sample of 258 Greek-speaking participants and identified 153 relationship problems. Study 2 used quantitative research methods on a sample of 783 Greek-speaking participants and classified them into 14 broader categories. The most common problems were a poor sex life, followed by incompatibility and neglect. Other common problems included a partner’s bad character, fear of abandonment, and lack of shared fun and recreation. Lack of loyalty and respect, disagreement over family planning, and privacy invasion were the least common problems in our sample. Both sexes reported similar problems, while the length of the relationship was not significantly associated with the presence of different relationship problems. Additionally, participants’ age, children, cohabitation, and relationship status were associated with some of the identified relationship problems.

**Supplementary Information:**

The online version contains supplementary material available at 10.1007/s12110-025-09489-7.

## Introduction

Maintaining an intimate relationship is not an easy endeavor, as evidenced by the high incidence of divorce (Cherlin, 2009; Raley & Sweeney, 2020; Stevenson & Wolfers, 2007) and singlehood (Apostolou et al., 2023a, b; Klinenberg, 2012). The purpose of the current research is to identify the different problems people face in their intimate relationships and to categorize them into broader categories. It also aims to examine whether individual differences, such as biological sex and years in the relationship, are associated with different relationship problems. The nature of these problems can be understood within an evolutionary theoretical framework, which we will discuss next.

## The Nature of Relationship Problems

### What are Lasting Intimate Relationships Good for?

To understand the nature of relationship problems, we need to understand the nature of intimate relationships, specifically addressing why people form lasting intimate relationships in the first place. In evolutionary terms, there are several important survival and reproductive benefits (jointly termed fitness benefits) to being in a lasting intimate relationship. More specifically, humans are a sexually reproducing species, which means that to procreate, people need access to the reproductive capacity of the opposite sex. However, sexual access alone is not sufficient, as human children require considerable, prolonged, and reliable parental investment to have a good chance of reaching sexual maturity. Such investment is difficult for one parent to bear, necessitating long-term collaboration between parents (Hawkes et al., 1989; Kim et al., 2012). Thus, individuals can derive considerable reproductive benefits by forming a lasting intimate relationship in which children can be born and raised (Lancaster & Lancaster, 1987).

Individuals can also provide substantial material resources to their intimate partners, which can substantially increase the latter’s chances of survival (Buss, 2016). These resources are especially important in the pre-industrial context, where there is no welfare state, and people rely on their partners and family in times of need. Moreover, intimate partners provide non-material benefits, including advice and emotional support during challenging times (Apostolou et al., 2023). They can also offer physical protection, which is crucial in pre-industrial contexts where protection institutions such as the police are absent (Buss, 2016). Overall, lasting intimate relationships are associated with considerable fitness benefits, translating into substantial selection pressures shaping the human mind to seek and establish long-term intimate relationships. Consequently, most people across different times and cultural settings form lasting intimate relationships, often referred to as marriage (Coontz, 2005). These relationships are frequently characterized by problems, the nature of which we will examine next.

### What is a Relationship Problem?

To summarize the argument above, people can derive considerable fitness returns from an intimate relationship, namely reproductive, material, and non-material benefits. We argue that any factor compromising the fitness people gain from an intimate relationship, predominantly by affecting these domains, would be interpreted as a relationship problem. More specifically, people would have evolved cognitive mechanisms that interpret any factors or situations compromising the fitness derived from the relationship as a problem—i.e., a matter or situation regarded as harmful that needs to be addressed and overcome. In this way, corrective action would be initiated. For instance, infidelity can severely compromise people’s fitness, as they share their partners’ resources with or risk losing them to others (Buss, 2000), so they would tend to interpret it as a relationship problem that demands corrective action. Such corrective actions might include threatening partners with consequences to motivate them to stop cheating or ending the relationship to find a more faithful partner. If infidelity were not interpreted as a problem, people would not think they need to do something about it, and thus they would not take action to contain their fitness losses.

This argument is not limited to actual but also to potential threats to the fitness people derive from a relationship. For example, if one partner is cheating, this constitutes an actual threat to the fitness the legitimate partner derives from the relationship. Nevertheless, if a partner does not actually cheat but has traits such as very attractive looks or a history of cheating, there is a high chance of future infidelity (Knopp et al., 2017; Ma-Kellams et al., 2017). Accordingly, in such a scenario, possible infidelity would be interpreted as a relationship problem. Moreover, a factor that can actually or potentially compromise fitness needs to be detected first to be interpreted as a problem. For instance, if individuals are unfaithful but successfully hide their infidelity from their partners, the latter will not consider infidelity a problem in their relationship. Overall, we define relationship problems as any perceived factors or situations that can actually or potentially compromise the fitness people derive from an intimate relationship. We will discuss some common factors or relationship problems next.

## Common Relationship Problems

One main factor that can compromise fitness is having a partner who does not score well in traits that predict the capacity to have and raise children and provide material and non-material benefits to one’s partner. Personality plays a central role here. For instance, hardworking individuals are more likely than lazy ones to generate the resources necessary to support their family, while kind and understanding individuals are more likely than selfish and unkind ones to divert their resources to their children and intimate partners (see also Buss & Hawley, 2010). Similarly, aggressive individuals are more likely to harm their children and partners than non-aggressive ones (Buss, 2000). In this respect, having a partner with traits such as laziness, unkindness, and aggressiveness could considerably compromise the fitness people can derive from the relationship, and thus, it would be interpreted as a relationship problem.

If a factor compromises fitness, it would be considered a problem, i.e., something the individual needs to address. This often involves the termination of the relationship, which means that research on divorce could reveal what people consider a relationship problem. Accordingly, studies on divorce find abusive behavior, unkind disposition, and laziness to be common reasons for marriage termination (Amato, 2010; Cherlin, 2009). Other situational factors, including infertility, poor health, and poor economic situation, can also affect the three domains. For instance, poor health could compromise individuals’ capacity to provide for their family and negatively affect their fertility. Accordingly, financial reasons constitute a common reason for divorce (Amato, 2010). Infertility and a partner’s poor health have also been reported as reasons, but much less frequently (Apostolou et al., 2019). In a different line of research, Apostolou and Wang (2020; 2021) examined what kept people from maintaining an intimate relationship. They identified 13 such factors, including partners being physically abusive.

Another major factor that can compromise fitness is incompatibility. More specifically, deriving fitness benefits from an intimate relationship requires effective cooperation between intimate partners, which, in turn, requires some degree of compatibility. Yet, partners may have traits that make them incompatible. For instance, being introverted or extroverted has no effect on the capacity to have family or to provide for it, but one partner being introverted and the other extroverted could potentially make them incompatible, which could compromise their capacity to cooperate effectively toward achieving fitness-increasing goals. As such, people would interpret a lack of compatibility as a relationship problem. Consistent with this argument, incompatibility is one of the most frequent reasons for getting a divorce (Amato, 2010; Amato & Previti, 2003).

By forming a lasting intimate relationship, people can increase their fitness considerably, but they can increase it even more by forming parallel intimate relationships. In particular, men can have additional offspring with, and women can secure material resources from extra-pair mates (Buss, 2000). Additionally, by engaging in extra-pair mating, individuals can probe future long-term partners in case their current ones abandon them, become incapacitated, or die (Buss et al., 2017). Extra-pair mating can potentially increase the fitness of the perpetrator considerably but decrease the fitness of the legitimate partner considerably. In particular, legitimate partners risk losing their mates and their investment to others. Men, in particular, risk raising other men’s children, believing they are their own, while both men and women risk contracting a sexually transmitted disease (Buss, 2000). Infidelity would then be considered a major relationship problem, and it is no surprise that it frequently leads to divorce (Amato & Previti, 2003; Apostolou et al., 2019). Similarly, Apostolou and Wang (2020; 2021) found partners’ infidelity, as well as not being monogamous, to be factors that prevented people from maintaining an intimate relationship.

The potentially high fitness costs of infidelity for the legitimate partner translate into strong selection pressures shaping mechanisms that would protect people from their partners’ infidelity, with romantic jealousy being the main one (Buss & Haselton, 2005). Hints of possible infidelity would trigger romantic jealousy, a negative emotion that people do not want to experience, motivating them to take corrective action, such as finding out whether an intimate partner is actually cheating (Apostolou & Antonopoulou, 2022). Nevertheless, romantic jealousy frequently triggers without the intimate partner cheating or intending to do so. One reason is that it may be too late to protect one’s fitness if a partner has already been involved in an extra-pair relationship. It could work better to prevent individuals from engaging in extra-pair relationships in the first place, and jealousy has evolved to do so (Buss, 2000).

More specifically, people feel jealous of their partners, which motivates them to take actions such as mate guarding, spying on their partners, threatening their partners with consequences, and so on (Buss & Shackelford, 1997), reducing their partners’ capacity to form extra-pair relationships. Jealousy can protect the fitness of one partner at the cost of the fitness of the other. To begin with, individuals who have jealous partners would face difficulty in having extra-pair relationships, while they will lose some of their freedom to pursue other fitness-increasing goals, such as having an extensive social network. In addition, jealousy can lead to bursts of violent behavior against one’s partner that can escalate to serious injury or even homicide (Buss, 2000). Romantic jealousy is then common in intimate relationships (Buss, 2016) frequently leading to divorce (Amato, 2010; Amato & Previti, 2003).

## The Current Study

When in an intimate relationship, people are confronted with problems. Yet, to the best of our knowledge, the existing literature lacks a comprehensive study on intimate relationship problems. The literature on divorce provides some evidence about these problems, but it is not adequate, as many of these problems are not likely to lead to divorce or may be severe enough to prevent people from marrying (and thus, divorcing) their partners in the first place. Additionally, many studies focus on individual problems such as jealousy (see Harris, 2003), which are insufficient for capturing the full range of problems people face in their relationships, as this was not their purpose. Apostolou and Wang (2020; 2021) focused on examining the factors that constrain people from maintaining an intimate relationship. Yet, these factors can reduce individuals’ fitness by making it difficult for them to maintain an intimate relationship, but they do not necessarily reduce the total fitness they receive from the relationship to classify as a relationship problem. For instance, Apostolou and Wang (2021) identified working long hours as a common constraint. Yet, a partner spending considerable time at work may actually increase the fitness people derive from the relationship, as it makes more resources available.

To address this gap in the literature, the current research employed a mixed-methods approach, where Study 1 used a combination of qualitative research methods to identify the different problems people face in an intimate relationship, and Study 2 employed quantitative research methods to classify these problems into broader categories. Our hypothesis is that people will identify as problems those factors that either actually or potentially reduce the fitness they derive from their intimate relationships.

In the present research, we aimed also to investigate individual differences in relationship problems. More specifically, factors such as years spent in a relationship, whether the couple is married or cohabitating, and the presence of children are likely to predict relationship problems, which we intend to examine. For instance, in the early stages of a relationship, individuals may face different challenges compared to the mature stages. It is also possible that in the early stages, people experience stronger romantic love than in later stages, which may lead them to downplay relationship issues. Furthermore, cohabitating may introduce new problems, such as domestic chores, while simultaneously reducing others, like infrequent interaction with one’s partner. Additionally, raising children demands considerable resources, including time and money, which can create strain in the relationship if these resources are not allocated towards maintaining it. On the other hand, having children may increase partners’ commitment to the relationship, leading them to downplay certain problems.

## Study 1

### Method

#### Participants

The study took place at a private university in the Republic of Cyprus and received approval from the institution’s ethics board. Participants were recruited through social media platforms (Facebook and Instagram) targeting individuals residing in Greece and the Republic of Cyprus, as well as through promotion to students and colleagues. The sole requirement for participation was a minimum age of 18 years. Participation was voluntary, with no monetary or other incentives offered.

##### Open-Ended Questionnaires

To ensure validity, we excluded participants who had never been in a relationship. The final sample included 218 Greek-speaking individuals (107 women, 111 men). The average age for women was 31.7 years (*SD* = 10.7), and for men, it was 33.1 years (*SD* = 11.2). Regarding relationship status, 70.8% were in an intimate relationship, 22.3% were single, and 6.9% indicated their relationship status as “other.”

##### In-Depth Interviews

The sample comprised 40 Greek-speaking individuals (20 women, 20 men) who were in an intimate relationship at the time of the study. The average age for women was 35.1 years (SD = 8.6), and for men, it was 34.9 years (SD = 7.5). In terms of relationship status, 68.4% were married, and 31.6% were in a relationship.

#### Materials

##### Open-Ended Questionnaires

The study was conducted in Greek, using Google Forms, and administered online. It consisted of two parts. The first part asked participants to “Write down some problems you currently face or have faced in the past, in your current or previous relationships,” with space provided for their responses. The second part collected demographic information, including sex, age, and relationship status. Participants were also asked if they had ever been in an intimate relationship in the past (yes, no).

#### In-Depth Interviews

The interviews were conducted in a lab on the university premises. Initially, participants were asked to indicate their age and relationship status. They were then asked to discuss problems they faced in their current and previous relationships, as well as issues faced by other couples they knew. The interviews lasted approximately 40 min on average.

### Analysis and Results

For data analysis, we enlisted two independent graduate students (one male and one female). Each assistant was instructed to review responses and create a list of the different problems that participants indicated that they faced in their relationships. They were further instructed to exclude responses with unclear or vague wording. Moreover, if a response included more than one issue (e.g., My boyfriend has a bad temper and he talks bad to me), the assistants were asked to add each issue separately in their list (i.e., bad temper, talks bad to me). They were also instructed to exclude responses with unclear or vague wording. After processing approximately 30% of the responses, the assistants compared their lists and then proceeded to process the remaining responses. Two lists of problems, one from each assistant, were produced and compared. Agreement was found for most items. In cases of disagreement, the authors were consulted, and a final list of actions was agreed upon by all parties involved. A total of 153 relationship problems were identified and are presented in Table 1 and in the Appendix.

**Table 1:** The relationship problems identified in study 1 and their classification into broader categories in study 2

Relationship problems Problems		Factor loadings	Cronbach’s α
**Incompatibility**			0.97
Different ways to see things		0.590	
Different needs		0.561	
Different priorities		0.526	
We want different things from the relationship		0.523	
Different views on the purpose of the relationship		0.490	
Different values		0.477	
Incompatibility in our characters		0.475	
Different interests with my partner		0.425	
My partner does not understand my needs and desires		0.386	
The relationship takes up a lot of my time		0.378	
My partner does not meet my expectations		0.376	
Lack of understanding		0.366	
Lack of consensus		0.355	
No clear boundaries are set in our relationship		0.350	
I force myself to maintain the relationship		0.325	
My partner does not consider my needs		0.316	
Lack of communication		0.301	
**Privacy invasion**			0.93
He/she goes through my emails/messages		0.653	
He/she searches my mobile		0.620	
He/she searches my things		0.515	
He/she tracks my social media activity		0.490	
**Fear of abandonment**			0.90
I am worried that my partner may leave me		− 0.764	
Fear that my partner will cheat on me		− 0.739	
Intense jealousy on my part		− 0.703	
I feel insecure that I am not good enough for him/her		− 0.631	
Lack of trust in my partner		− 0.446	
I don’t know what my partner really wants		− 0.346	
I have hard time understanding my partner’s needs		− 0.335	
**Lack of loyalty and respect**			0.94
Infidelity		0.461	
Physical abuse		0.441	
He/she is flirtatious with others		0.424	
Keeps in touch with his/her ex		0.413	
He makes negative comments about me in front of others		0.396	
My partner criticizes me in front of others		0.373	
Lies		0.300	
**Partner’s messiness**			0.87
Doesn’t tidy up his/her things		0.851	
He/she is messy		0.816	
We disagree about the cleanliness of the house		0.584	
We disagree on the house chores - who will do what		0.548	
He/she is lazy		0.375	
Forgets to do the things I tell him/her to do		0.350	
**Partner’s bad character**			0.98
His/her bad temper		− 0.706	
Has difficulty controlling his/her nerves		− 0.656	
He/she is irritable		− 0.639	
Tensions		− 0.573	
Fights		− 0.550	
He/she talks bad to me		− 0.520	
Nagging		− 0.508	
Bad behavior from my partner		− 0.469	
Always insists to his/her point of view		− 0.464	
He/she is selfish		− 0.453	
He/she criticizes me all the time		− 0.391	
Lack of respect		− 0.386	
Manipulative behavior from my partner		− 0.386	
He/she doesn’t back down		− 0.369	
Emotional abuse		− 0.360	
He/she degrades me		− 0.359	
Emotional blackmail		− 0.351	
He/she nags me		− 0.342	
He/she is emotionally unstable		− 0.333	
Threats from my partner		− 0.309	
My partner has psychological problems		− 0.308	
**In-law and social circle conflicts**			0.86
I don’t get along with his/her relatives		0.899	
Incompatible relatives		0.856	
My partner’s family is interfering with our relationship		0.702	
He/she doesn’t get along with my relatives		0.543	
He/she doesn’t get along with my friends		0.344	
I don’t get along with his/her friends		0.318	
**Bad sex life**			0.95
Lack of sex		0.958	
The frequency of sex		0.881	
Bad love life		0.830	
My partner does not satisfy me sexually		0.828	
Incompatibility in sex		0.720	
Lack of passion		0.678	
Routine		0.370	
Boredom		0.368	
Growing apart		0.307	
**Partner’s neglect of health and appearance**			0.90
He/she does not take care of his/her health		0.713	
He/she does not pay attention to his/her diet		0.666	
He/she does not take care of himself/herself		0.654	
I am worrying about my partner’s health		0.595	
He/she does not care about his/her appearance		0.538	
He/she does not take care of his/her hygiene		0.475	
My partner has health problems		0.471	
My partner’s appearance does not satisfy me as it did at first		0.304	
**Neglect**			0.96
He/she doesn’t give me enough time		0.501	
My partner does not show his/her feelings		0.436	
Neglect by my partner		0.426	
Lack of tenderness		0.419	
Indifference from my partner		0.413	
He/she doesn’t have me as a high priority		0.402	
He/she is distant		0.399	
My partner ignores me		0.374	
Little quality time with my partner		0.369	
He/she doesn’t care about me		0.366	
He/she avoids talking about our problems		0.306	
My partner spends long hours at work		0.301	
**Disagreement over family planning**			0.90
Disagreement about whether to have more children		0.556	
Parenting disputes		0.535	
Disputes about the responsibilities each of us has for raising children		0.533	
Disagreement about whether to have children		0.472	
My partner does not spend enough time with the children		0.446	
My partner does not treat our children well		0.390	
**Lack of shared fun and recreation**			0.84
We don’t go out together often		0.537	
We don’t agree on how to have fun		0.516	
We don’t do things together		0.408	
He/she doesn’t feel like going out		0.389	
We disagree on what to eat		0.372	
**Partner’s jealousy**			0.96
He/she won’t let me go out alone		0.565	
Jealousy		0.544	
He/she deprives me of my freedom		0.534	
He keeps checking up on me		0.534	
Intense jealousy on the part of my partner		0.530	
He/she won’t let me have friends of the opposite friend		0.524	
My partner’s clinging to me		0.493	
Possessiveness by my partner		0.461	
Suspicions		0.387	
Lack of trust from my partner		0.386	
He/she oppresses me		0.377	
Lack of personal space		0.361	
He/she spies on me when I go out		0.327	
**Wasteful with money**			0.86
He/she spends more than our potential		0.676	
He/she is wasteful		0.642	
He/she has addictions		0.405	
Financial problems		0.364	
He/she is addicted to gambling		0.339	

## Study 2

### Methods

#### Participants

To obtain a diverse sample, we employed different recruitment strategies. We distributed the study link to students and colleagues, asking them to share it further. Additionally, we promoted the link on social media platforms, including Facebook and Instagram, targeting individuals residing in Greece and the Republic of Cyprus. We also created a QR code linking to our study and recruited participants in cafés and malls in Nicosia, Republic of Cyprus. The requirements for participation included a minimum age of 18 years and being in an intimate relationship at the time of the study. Participation was voluntary, with no monetary or other incentives offered.

The initial sample consisted of 997 participants. However, we excluded responses from participants who indicated their relationship status as “single” or “other,” responses that were less than 80% complete, and responses from participants who failed the attention measures. The final sample comprised 783 Greek-speaking participants (436 women, 344 men, and three participants who did not indicate their sex). The mean age for women was 34.8 years (*SD* = 11.1), and for men, it was 37.9 years (*SD* = 12.6). Additionally, 56.3% of participants were in a relationship but not married, and 43.8% were married. Furthermore, 55.1% had children, and 44.9% did not, while 66.8% were cohabiting with their partners, and 33.2% were not. Participants had been in their relationships for an average of 9.6 years (*SD* = 10.4).

#### Materials

The study was conducted online, with the questionnaire in Greek, created using Google Forms, and consisting of two parts. In the first part, participants were presented with the following scenario: “Below you will find a number of problems that people may face in their romantic relationships. Indicate to what extent each of the following is a problem in your current relationship.” They were then asked to rate the 153 items identified in Study 1 on a scale from 1 (Not a problem at all) to 5 (A very serious problem). The order of item presentation was randomized for each participant. The second part collected demographic information, including biological sex, age, relationship status (single, in a relationship, married, other), and years in the current relationship. Participants were also asked whether they had children (yes/no), and whether they were cohabiting with their partners (yes/no).

#### Data Analysis

To classify the 153 items into broader factors or problems, we applied exploratory factor analysis using principal axis factoring for factor extraction with direct oblimin rotation. To determine the number of extracted factors, we employed the Kaiser criterion, retaining all factors with an eigenvalue of at least one. Additionally, to decide how many items to retain in each factor, we used a cutoff point of 0.30. We also conducted a series of MANCOVA tests to identify significant effects. Specifically, for each identified problem, the items that composed it were entered as dependent variables. Sex, relationship status, children, and cohabiting status were entered as independent categorical variables, while age and years in the current relationship were entered as continuous independent variables. This analysis was performed for each problem identified.

## Results

The Kaiser criterion suggested a 14-factor solution. How the extracted factors correlate with each other can be seen from the correlation matrix in the supplementary material. The KMO measure was 0.98, indicating that our sample was highly suitable for exploratory factor analysis. Initial analysis revealed that some items had factor loadings below the 0.30 threshold. To address this issue, we conducted a revised analysis by excluding the item with the lowest factor loading and repeated the procedure until all items had loadings of at least 0.30. In total, 27 items were removed, and they are listed in the Appendix. The internal consistency, as measured by Cronbach’s alpha, ranged from 0.84 to 0.97 (Table 1).

The first relationship problem to emerge was “Incompatibility,” where participants were troubled by seeing things differently, having different needs, wants, and priorities with their partners. Lack of understanding and perceived incompatibility of characters were additional facets of this dimension. The second relationship problem to emerge was “Privacy Invasion,” where participants indicated their partners searching their things, emails, and mobiles as problems in their relationship. The “Fear of Abandonment” was the next relationship problem to emerge, where participants indicated that they feared their partners would leave or cheat on them, and that they were not good enough for them. Experiencing intense jealousy and mistrust for their partners, and failing to understand their wants and needs were additional facets of this problem. In the “Lack of Loyalty and Respect” problem, individuals were troubled by their partners’ infidelity and abusive behavior, as well as negative comments and criticism in front of others. Participants were also troubled by their “Partner’s Messiness” and their disagreement about the cleanliness of the house. Laziness also loaded here, which we interpret as laziness specifically related to cleaning the house rather than laziness in general.

Moreover, participants indicated their “Partner’s Bad Character” as another relationship problem. In particular, they were troubled by their partners’ difficulty in controlling their nerves, being irritable, selfish, inflexible, disrespectful, emotionally unstable, and manipulative. These negative traits presumably lead to fights and tensions, which also loaded onto this factor. In the “In-law and Social Circle Conflicts” problem, participants indicated not getting along with their partners’ relatives and friends or their partners not getting along with participants’ relatives and friends as issues in their relationship. In the “Bad Sex Life” problem, participants reported the frequency and quality of sex to be issues in their relationship. Boredom and routine were two facets of this problem. Furthermore, participants indicated a relationship problem to be their “Partner’s Neglect of Health and Appearance.” That is, they were worried by their partners neglecting their health, putting on extra weight, and not taking care of themselves. In the “Neglect” relationship problem, individuals had issues with their partners neglecting them, not showing them any tenderness, being indifferent and distant, and ignoring them.

In the “Disagreement over Family Planning” problem, participants indicated as relationship issues disagreements with their partners on whether they would have children or more children, on how to raise their children, and how to split responsibilities in raising children. One facet of this problem was partners not spending enough time with or not treating their children well. In the “Lack of Shared Fun and Recreation” problem, relationship issues included not doing things with one’s partner and disagreement on how to have fun. One further relationship problem was “Partner’s Jealousy.” Here, participants were troubled by their partners being jealous, not letting them go out alone, depriving them of their freedom, being possessive, suspicious, and not trusting them. The final relationship problem to emerge was individuals’ partners being “Wasteful with Money.” Addictions such as gambling leading to money being wasted also loaded here.

Moreover, we calculated means and standard deviations for each identified problem, and we have placed them in a hierarchical order in Table 2. Note that, the means for each sex are the averages split by sex and not estimated marginal means. We can see that the “Bad Sex Life” was the most common problem, followed by “Incompatibility,” “Neglect,” and “Partner’s Bad Character.” At the bottom of the hierarchy were “Lack of Loyalty and Respect,” “Disagreement over Family Planning,” and “Privacy Invasion.”

**Table 2:** Mean scores, sex and relationship status effects in Study 2

Problems		Overall	Women	Men	Sex		Relationship status	
		Mean (SD)	Mean (SD)	Mean (SD)	*p-*value	η_p_^*2*^	*p-*value	η_p_^*2*^
Bad sex life		2.55 (1.27)	2.57 (1.26)	2.54 (1.29)	0.004	0.032	0.026	0.024
Incompatibility		2.54 (1.21)	2.65 (1.24)	2.42 (1.17)	0.015	0.045	0.028	0.042
Neglect		2.50 (1.27)	2.63 (1.28)	2.34 (1.26)	0.289	0.020	0.458	0.027
Partner’s bad character		2.45 (1.25)	2.48 (1.29)	2.41 (1.21)	0.015	0.054	0.001	0.066
Fear of abandonment		2.37 (1.17)	2.46 (1.18)	2.25 (1.17)	0.269	0.012	0.749	0.006
Lack of shared fun and recreation		2.22 (1.03)	2.30 (1.07)	2.13 (0.97)	0.030	0.018	0.307	0.009
Partner’s messiness		2.06 (1.01)	2.18 (1.05)	1.90 (0.92)	0.066	0.017	0.009	0.024
Wasteful with money		2.03 (1.11)	2.09 (1.15)	1.95 (1.06)	0.017	0.019	0.099	0.013
Partner’s jealousy		2.00 (1.13)	2.00 (1.17)	2.01 (1.09)	0.583	0.016	0.006	0.041
Partner’s neglect of health and appearance		1.94 (0.97)	1.99 (0.99)	1.89 (0.94)	0.416	0.012	0.368	0.012
In-law and social circle conflicts		1.90 (0.99)	1.91 (1.00)	1.88 (0.98)	0.974	0.002	0.012	0.023
Lack of loyalty and respect		1.88 (1.19)	1.90 (1.19)	1.85 (1.19)	0.573	0.008	0.001	0.034
Disagreement over family planning		1.81 (1.08)	1.82 (1.11)	1.81 (1.05)	0.363	0.010	0.010	0.024
Privacy invasion		1.65 (1.13)	1.61 (1.12)	1.72 (1.13)	0.051	0.013	< 0.001	0.031

### Significant Effects

In total, 14 MANCOVA tests were performed, and to reduce the probability of committing a Type I error, we applied Bonferroni correction for alpha inflation, setting the alpha level to 0.003 (0.050/14). Thus, the reader should not consider as significant any results above this level. From Table 2, we can see that a significant main effect of sex was observed for the “Bad Sex Life.” Yet, the means were almost identical. For the items referring specifically to sex, namely “Lack of Sex,” “The Frequency of Sex,” “Bad Love Life,” “My Partner Does Not Satisfy Me Sexually,” men gave higher scores than women, while for “Lack of Passion,” “Routine,” “Boredom,” and “Growing Apart,” women gave higher scores than men. Yet, we need to note that the effect size was small, indicating that the two sexes were very similar in this dimension.

Moreover, there was a significant main effect of relationship status on “Partner’s Bad Character,” with participants in a relationship giving higher scores (*M* = 2.63, *SE* = 0.10) than married participants (*M* = 2.47, *SE* = 0.26). Note that we have calculated here the marginal means, that is, the average scores across all levels of the independent variables in our analysis. Additionally, such an effect was observed for “Lack of Loyalty and Respect,” where participants in a relationship gave higher scores (*M* = 2.12, *SE* = 0.10) than married participants (*M* = 1.97, *SE* = 0.24). This effect was also observed for “Privacy Invasion,” where participants in a relationship gave higher scores (*M* = 1.90, *SE* = 0.10) than married participants (*M* = 1.60, *SE* = 0.23).

As we can see from Table 3, there was a significant main effect of children on “Partner’s Bad Character,” with participants who had children giving higher scores (*M* = 2.65, *SE* = 0.18) than participants who did not have children (*M* = 2.45, *SE* = 0.19). This was also the case for “Lack of Shared Fun and Recreation,” with participants who had children giving higher scores (*M* = 2.44, *SE* = 0.15) than participants who did not have children (*M* = 2.15, *SE* = 0.16). A similar effect was found for “Partner’s Jealousy,” with participants who had children giving higher scores (*M* = 2.41, *SE* = 0.17) than participants who did not have children (*M* = 1.99, *SE* = 0.17). This was also the case for “Lack of Loyalty and Respect” yet, the means were very similar. That is, participants who had children gave similar scores (*M* = 2.04, *SE* = 0.17) to participants who did not have children (*M* = 2.06, *SE* = 0.18). Looking at the subcomponents of this factor, for “Infidelity,” participants who had children gave higher scores (*M* = 1.99, *SE* = 0.21) than participants who did not have children (*M* = 1.74, *SE* = 0.22). This was also the case for “Physical Abuse,” with participants who had children giving higher scores (*M* = 1.78, *SE* = 0.18) than participants who did not have children (*M* = 1.45, *SE* = 0.19). On the other hand, for “He/She Is Flirtatious with Others,” participants who had children gave lower scores (*M* = 1.89, *SE* = 0.21) than participants who did not have children (*M* = 2.35, *SE* = 0.21). For the rest of the items, the scores of parents and non-parents were similar.

**Table 3:** Children, cohabiting, age, and years in relationship effects in study 2

Problems		Children		Cohabiting		Age		Years in relationship	
		*p-*value	η_p_^*2*^	*p-*value	η_p_^*2*^	*p-*value	η_p_^*2*^	*p-*value	η_p_^*2*^
Bad sex life		0.065	0.021	0.373	0.012	0.080	0.020	0.221	0.015
Incompatibility		0.054	0.039	0.456	0.024	(+) 0.001	0.058	0.309	0.028
Neglect		0.021	0.034	0.293	0.020	0.330	0.019	0.711	0.013
Partner’s bad character		< 0.001	0.084	0.032	0.050	(+) < 0.001	0.080	0.426	0.031
Fear of abandonment		0.234	0.013	0.749	0.006	(+) 0.003	0.030	0.025	0.022
Lack of shared fun and recreation		< 0.001	0.043	0.172	0.011	0.859	0.003	0.400	0.007
Partner’s messiness		0.164	0.013	0.067	0.017	0.277	0.011	0.181	0.012
Wasteful with money		0.065	0.015	0.089	0.013	0.063	0.015	0.016	0.019
Partner’s jealousy		< 0.001	0.082	0.017	0.037	(+) 0.002	0.045	0.102	0.028
Partner’s neglect of health and appearance		0.125	0.018	0.759	0.007	0.013	0.027	0.064	0.021
In-law and social circle conflicts		0.153	0.013	0.001	0.032	0.221	0.012	0.198	0.012
Lack of loyalty and respect		< 0.001	0.005	< 0.001	0.047	0.046	0.020	0.047	0.020
Disagreement over family planning		0.016	0.023	0.461	0.008	0.275	0.011	0.267	0.011
Privacy invasion		0.378	0.006	0.017	0.017	0.816	0.002	0.606	0.004

Note. The signs in parenthesis indicate the direction of the relationship

Also from Table 3, we can see that there was a significant main effect of cohabiting only for “Lack of Loyalty and Respect,” where participants who cohabited with their partners gave significantly lower scores (*M* = 1.89, *SE* = 0.08) than participants who did not (*M* = 2.23, *SE* = 0.25). Furthermore, there was a significant main effect of age on four problems. As indicated by the effect size, the largest one was over “Partner’s Bad Character,” where the relationship was positive, indicating that older participants gave higher scores. We can also see that there was no significant main effect of years in a relationship for any of the identified problems.

## Discussion

In the current research, using a mixed-methods approach, we identified 14 relationship problems. The most common ones included a bad sex life, followed by incompatibility and neglect. Partner’s bad character, fear of abandonment, and lack of shared fun and recreation were other common problems. Lack of loyalty and respect, disagreement over family planning, and privacy invasion were the least common problems in our sample. We also found that the two sexes reported similar problems, while the length of the relationship was not significantly associated with the presence of different relationship problems. On the other hand, participants’ age, children, cohabitation, and relationship status were significantly associated with several relationship problems.

The most common problem in our sample was a bad sex life. This problem may reflect incompatibility in sex; for instance, partners may not share similar tastes, or a partner may have a sexual difficulty such as low libido. Nonetheless, it can also reflect other problems. For instance, people not being satisfied with their partners because they are jealous, aggressive, or lazy may result in not wanting to have sex with them. This being the case, a bad sex life is a problem arising from sexual factors, but it also reflects other relationship problems, which can explain why it was the most common in our sample.

To be lasting, an intimate relationship requires effective cooperation between intimate partners, which in turn requires some degree of compatibility. For instance, studies on divorce indicate that incompatibility is a major reason why intimate relationships collapse (Amato, 2010). Here, one of the most common problems in our sample was incompatibility, which came second in the hierarchy. Yet, the problem of incompatibility is reflected in other identified problems. In particular, in the “Bad Sex Life,” participants indicated that they were sexually incompatible with their partners, while in the “Lack of Shared Fun and Recreation,” participants indicated incompatible leisure preferences. The “Partner’s Messiness” problem also reflects different views about keeping things tidy and clean.

We have argued that people can derive considerable material and non-material benefits from their intimate partners, which is not going to happen if the latter are indifferent and neglectful of the former. Accordingly, “Neglect” from one’s intimate partner emerged as a problem in our study. Moreover, character traits such as bad temper, selfishness, and irritability, reflected in the “Partner’s Bad Character” problem, would make effective cooperation between partners difficult. Additionally, having intimate partners who are “Wasteful with Money” reduces the monetary resources available for one’s family, and thus, the fitness people derive from the relationship.

One reason that an intimate relationship can be compromised is that people’s partners have a mate value higher than their own. For instance, if individuals have partners who are much more attractive than they are, they risk losing them to others. To prevent this compromise in their fitness, this asymmetry in mate value may trigger negative emotions such as jealousy or fear of abandonment that could motivate preventive action such as mate guarding (see Buss & Shackelford, 1997). Accordingly, the “Fear of Abandonment” problem emerged, where people would feel insecurity in their relationship, fear that they are not good enough for their partners who are likely to leave them, and jealousy. It could also be the case that some individuals are predisposed to experience high jealousy and fear of abandonment irrespective of the differential in fitness with their partners. This predisposition could potentially confer fitness benefits by keeping them vigilant and motivating them to exercise mating effort in keeping their partners.

Romantic jealousy increases fitness by increasing vigilance and by constraining partners’ freedom to act as they wish (which could potentially involve cheating) (Buss, 2000). Yet, the reduction in freedom also means a reduction in fitness, which makes people see a “Partner’s Jealousy” as a relationship problem. This issue is also reflected in the “Privacy Invasion” problem, where, most likely driven by jealousy, individuals search their partners’ things, emails, and mobile phones. This problem was the least common in our sample, one reason being that people may not frequently engage in such actions. Another reason could be that when they do it, they are careful to remain undetected.

Intimate partners who are incapacitated by disease or death cannot provide for their mates and children. Accordingly, when individuals neglect themselves, for instance by overeating, they do not harm only their fitness but also their partners’ fitness, who consequently interpret this to be a problem reflected in the “Partner’s Neglect of Health and Appearance.” Furthermore, partners usually come along with their friends and relatives. Thus, effective cooperation with intimate partners requires, to some degree, getting along with their family and friends. For instance, in ancestral societies throughout most of human evolution, when wealth was limited and social welfare systems were absent, parents-in-law likely played an important role in child-rearing and served as vital sources of protection and resources. In effect, conflicts with in-laws could have jeopardized one’s access to valuable resources and support for offspring, potentially diminishing the fitness benefits derived from the romantic relationship. The concern with poor relationship with a partner’s friends and relatives is reflected in the “In-law and Social Circle Conflicts” problem. Furthermore, one primary benefit people derive from an intimate relationship is to be able to have and raise children. It follows that if intimate partners do not wish to have children, this constitutes a major compromise in the fitness one derives from the relationship, as reflected in the “Disagreement over Family Planning” problem.

Having partners who are unfaithful or physically abusive can cause a substantial reduction in fitness, which is reflected in the “Lack of Loyalty and Respect” problem. We found that this was not a common problem people faced in our sample. One reason could be that most people do not cheat or physically abuse their partners, so this is not a common issue. Another reason is that infidelity is done in secrecy, so many of the participants in our sample may have unfaithful partners but may not be aware of it. Yet another reason could be that participants are less likely to share information on physical abuse or unfaithfulness as it is particularly sensitive topic. Moreover, infidelity and physical abuse have very high negative fitness consequences, which means that people who experience such problems would most likely end the relationship, so they would not be in our sample.

The latter argument invites some further interpretation of our findings. In particular, we found that a bad sex life, incompatibility, and neglect were the most common problems in our sample. One possible reason is that they may not be so fitness-impairing as to justify ending the relationship, increasing the probability of being present in our sample. For instance, having an intimate partner who is a bit more introverted than one is, or sees some aspects of life differently, or has different hobbies than one has, is likely to have a negative impact on the fitness people derive from the relationship to be interpreted as a problem, but not a big negative impact to justify ending the relationship on these grounds alone. Of course, what matters also is the degree of the problem. For instance, it is unlikely for people to find a partner who is perfectly compatible with them, so they will accept some degree of incompatibility. Yet, if there are major differences in how the partners see things, including the relationship, this is unlikely to lead to effective cooperation between them toward achieving common goals, as there are no common goals, so ending the relationship may be the best way.

This argument can also explain the low means we have recorded in our study. Given our five-point scale, all the means were below the ‘3’ midpoint, indicating that a substantial proportion of the participants in our sample either did not experience the identified problems or did not experience them to a degree that would affect their relationship considerably. The reason for this is that people who experienced many of these problems, or some of these problems to a degree that would considerably affect their relationship, would most probably not be in it, and thus, would not be in our sample. Yet, this is not a limitation of our study, but a simple fact that most people who are in an intimate relationship do not face severe problems, and that is why they continue being in it.

A significant sex difference was found for the “Bad Sex Life” problem, where men gave higher scores than women for the physical aspect (e.g., frequency of sex) while women gave higher scores for the emotional aspect (e.g., lack of passion). One possible reason is that men ascribe more importance to the physical aspect while women to the emotional aspect of sex (see Harris, 2003). Still, in all remaining dimensions, no significant sex differences were identified, indicating that men and women face similar relationship problems. Older participants were more likely to report problems such as partner’s bad character and incompatibility than younger participants. One possibility is that older people consider it more important for their partners to be compatible with them and have good personality traits, so they are more troubled if this is not the case than younger participants. One reason is that these traits are highly important for a harmonious relationship (Buss, 2016), which older people who have more relationship experience are more likely to be aware of than younger ones.

Married participants gave lower scores in the “Partner’s Bad Character,” the “Lack of Loyalty and Respect,” and the “Privacy Invasion” than participants in a relationship. One possible interpretation of this finding is that people consider these dimensions important for taking the relationship to the next level, so if these problems are not present, they are more likely to marry their partners. Additionally, for the “Lack of Loyalty and Respect” problem, participants who cohabited with their partners gave significantly lower scores than participants who did not. As in the case of marital status, one reason may be that participants who had unfaithful or abusive mates were unlikely to move in with them. Furthermore, participants who had children gave higher scores for their “Partner’s Bad Character” and the “Partner’s Jealousy” than participants who did not have children. One possibility is that traits such as bad temper and jealousy may interfere with raising children effectively (e.g., individuals may become abusive), and once individuals have children, they become more aware of them in their partners. It could also be the case that participants with children are potentially more likely to stay with their partners for the sake of their children and therefore could maintain the relationship despite “Partner’s Bad Character” and “Partner’s Jealousy”. Moreover, participants who had children gave higher scores on the “Lack of Shared Fun and Recreation” than participants who had no children. One likely reason is that people who have children have to devote more time to raising them, which is taken from the time they spend with their mates in recreational activities.

We were surprised that the years in a relationship were not significantly associated with relationship problems. There may be different reasons for the lack of such an effect. For instance, in the early stages of the relationship, people may hide some negative aspects of their character from their partners, or by being in love, tend to ignore the shortcomings of their partners. Yet, as the relationship progresses, these shortcomings would become apparent and/or people will stop ignoring them, so the more years people are in a relationship, the more problems they are likely to experience. On the other hand, people who experience limited problems in their relationship are more likely to remain in it, which means that years in a relationship constitute a proxy of how many problems people have: The more years people are in a relationship, the fewer problems they have. These effects cancel out; that is, in some relationships, one effect is occurring, while in others, the opposite effect is occurring, and thus these opposing effects could cancel each other out when looking at the overall data.

Apostolou and Wang (2020; 2021) identified 13 different factors, six of which closely match the factors we have identified here, including character issues, partners’ abusive behavior, infidelity, bad sex, children, and social circle issues. Still, the remaining seven factors, including partner’s clinginess, long-working hours, lack of effort, lack of personal time and space, fading away enthusiasm, not making compromises, and not being monogamous, did not have a close match to the factors we have identified here. As discussed in the introduction, one possible reason is that these factors could constrain people from keeping an intimate relationship, but they may not necessarily reduce the fitness they derive from an intimate relationship to classify as relationship problems. For instance, not being monogamous does not reduce the fitness one receives from the relationship unless one acts on it and the partner detects it.

Although most people will face relationship problems at some point in their lives, the existing literature on what these problems are and what predicts them is surprisingly limited. Given the complexity of this issue, many more studies are needed to understand the phenomenon thoroughly, which would benefit from a psychometric instrument designed to measure relationship problems. For our research, we developed such an instrument, ensuring its validity by directly asking individuals about the problems they faced in their relationships. However, the instrument we created is relatively lengthy, which may limit its practical use. Future studies could aim to develop a shorter version by selecting three or four items with the highest loading from each extracted factor. Researchers could then correlate the scores from the shorter instrument with those from the original and examine the degree of overlap.

In the current research, we aimed not only to provide a list of the problems people face in intimate relationships but also to understand their nature. From an evolutionary perspective, the brain has evolved to process information to generate behavior that increases fitness (Barkow et al., 1992). Using this perspective, we argue that people would interpret relationship-related factors that actually or potentially impair their fitness as problems, enabling them to take corrective action. This being the case, all intimate relationship problems could be reduced to actual or potential threats to fitness, a prediction that needs further investigation. Moreover, we need to consider that cognitive hardware has been shaped in an ancestral context, which is very different from the contemporary one. This mismatch between ancestral and modern conditions may result in inaccurate interpretations by the mind regarding what constitutes relationship problems in contemporary settings (see also Li et al., 2018). For instance, as discussed above, getting along with one’s relatives was critically important in the ancestral context, but it holds limited importance in the contemporary one. Yet, people interpret not getting along with their partners’ relatives as an important relationship problem. In this respect, the prediction should be refined: All intimate relationship problems could be reduced to actual or potential threats to fitness in the ancestral pre-industrial environment, and quite likely in the contemporary one as well. We should also mention that this theoretical argument could be employed in reverse: Any relationship-related factors that promoted people’s fitness in ancestral human societies—and most likely in contemporary ones—would be interpreted as a strength or advantage of the relationship, motivating people to keep it.

One limitation of the current research is that we employed self-report measures that are subject to biases, such as participants giving inaccurate answers. For instance, some people may not be willing to admit that they experience sexual problems in their relationship. Moreover, our research was based on non-probability samples, so our findings may not readily apply to the population. Furthermore, the research took place in the Greek cultural context, so its findings may not readily apply to other cultural settings. Accordingly, future studies need to attempt to replicate our findings in different cultures and examine differences and similarities individuals face. Additionally, in the present research, we did not measure sexual orientation, and future studies need to examine whether the identified problems also reflect the issues non-heterosexual couples face. Likewise, there are several variables we have not measured, such as education and income, that are likely to predict the relationship problems we have identified. Future studies should explore the effects of a more comprehensive list of factors on relationship problems.

Moreover, when exploratory factor analysis is used, the resulting factor structure is sensitive to the sample’s properties. Therefore, to increase our confidence that the observed structure accurately reflects the actual structure of relationship problems, additional studies using confirmatory factor analysis across different samples are needed.

Intimate relationships tend not to be problem-free, and in the current study, we have traced their roots in their negative effect on the fitness people derive from the relationship. We have identified several problems that plague intimate relationships and some factors that predict these problems. The complexity of the phenomenon requires, however, considerably more research in order to be better understood.

## Appendix

The items below were identified in Study 1, but were not included in the factor structure identified in Study 2.

1. He/she doesn’t ask my opinion before he/she does something.
2. Incompatible friends.
3. My partner has changed since we started dating.
4. He/she doesn’t appreciate what I do for him/her.
5. My partner’s behavior is not like it was at the beginning of the relationship.
6. My partner takes our relationship for granted.
7. My partner makes too many demands on me.
8. Fear of taking the relationship to the next level.
9. My partner questioning my feelings.
10. My partner’s strong dependence on me.
11. Lack of support from my partner.
12. He/she puts all the responsibility on me.
13. I find it difficult to be monogamous.
14. My family gets involved in our relationship.
15. We argue about the decoration of the house.
16. The relationship is getting in the way of my career/studies.
17. Distance - we stay away from each other.
18. My partner’s stinginess.
19. He/she is addicted to alcohol.
20. Not understanding of my career needs.
21. Lack of honesty.
22. He/she tries to change me.
23. He/she does not have a good job.
24. Lack of personal time.
25. My partner is always judging my appearance negatively.
26. My partner thinks I am inferior.
27. I am afraid that something bad will happen to my partner.

## Electronic Supplementary Material

Below is the link to the electronic supplementary material.

Supplementary Material 1

## Data Availability

All data are available here: https://osf.io/c79xg/?view_only=1a45648744694a62be17552fa1629a24.
