# Supplementary material for: The Nature, Taxonomy, and Contingencies of Intimate Relationship Problems
Source: Hum Nat. 2025 Mar 24;36(1):98–120. doi: 10.1007/s12110-025-09489-7 (PMC12058819; doi:10.1007/s12110-025-09489-7)
Supplement: Supplementary file 1 — Supplementary Material 1 [file 12110_2025_9489_MOESM1_ESM.docx]

**Supplementary Material**

We have correlated all the extracted factors with each other using Pearson’s product moment correlation. The produced correlation matrix is below.

| **Correlations** | | | | | | | | | | | | | | | |
| --- | --- | --- | --- | --- | --- | --- | --- | --- | --- | --- | --- | --- | --- | --- | --- |
|  | | Incompatibility | Privacy invasion | Fear of abandonment | Lack of loyalty and respect | Partner’s messiness | Partner’s bad character | In-law and social circle conflicts | Bad sex life | Partner's neglect of health and appearance | Neglect | Disagreement over family planning | Lack of shared fun and recreation | Partner’s jealousy | Wasteful with money |
| Incompatibility | Pearson Correlation | 1 | .531^**^ | .707^**^ | .675^**^ | .640^**^ | .870^**^ | .646^**^ | .795^**^ | .624^**^ | .853^**^ | .656^**^ | .785^**^ | .703^**^ | .650^**^ |
|  | Sig. (2-tailed) |  | <.001 | <.001 | <.001 | <.001 | <.001 | <.001 | <.001 | <.001 | <.001 | <.001 | <.001 | <.001 | <.001 |
|  | N | 756 | 751 | 751 | 746 | 747 | 725 | 749 | 748 | 743 | 736 | 726 | 750 | 737 | 749 |
| Privacy invasion | Pearson Correlation | .531^**^ | 1 | .524^**^ | .719^**^ | .494^**^ | .637^**^ | .591^**^ | .438^**^ | .591^**^ | .487^**^ | .691^**^ | .422^**^ | .802^**^ | .657^**^ |
|  | Sig. (2-tailed) | <.001 |  | <.001 | <.001 | <.001 | <.001 | <.001 | <.001 | <.001 | <.001 | <.001 | <.001 | <.001 | <.001 |
|  | N | 751 | 770 | 761 | 758 | 758 | 734 | 762 | 761 | 755 | 749 | 738 | 762 | 750 | 763 |
| Fear of abandonment | Pearson Correlation | .707^**^ | .524^**^ | 1 | .725^**^ | .463^**^ | .705^**^ | .551^**^ | .561^**^ | .490^**^ | .758^**^ | .554^**^ | .587^**^ | .582^**^ | .583^**^ |
|  | Sig. (2-tailed) | <.001 | <.001 |  | <.001 | <.001 | <.001 | <.001 | <.001 | <.001 | <.001 | <.001 | <.001 | <.001 | <.001 |
|  | N | 751 | 761 | 769 | 756 | 758 | 735 | 762 | 759 | 754 | 747 | 736 | 761 | 748 | 760 |
| Lack of loyalty and respect | Pearson Correlation | .675^**^ | .719^**^ | .725^**^ | 1 | .573^**^ | .780^**^ | .659^**^ | .583^**^ | .664^**^ | .708^**^ | .762^**^ | .578^**^ | .737^**^ | .751^**^ |
|  | Sig. (2-tailed) | <.001 | <.001 | <.001 |  | <.001 | <.001 | <.001 | <.001 | <.001 | <.001 | <.001 | <.001 | <.001 | <.001 |
|  | N | 746 | 758 | 756 | 764 | 754 | 730 | 757 | 754 | 749 | 744 | 733 | 757 | 744 | 757 |
| Partner’s messiness | Pearson Correlation | .640^**^ | .494^**^ | .463^**^ | .573^**^ | 1 | .621^**^ | .567^**^ | .578^**^ | .639^**^ | .587^**^ | .591^**^ | .606^**^ | .542^**^ | .640^**^ |
|  | Sig. (2-tailed) | <.001 | <.001 | <.001 | <.001 |  | <.001 | <.001 | <.001 | <.001 | <.001 | <.001 | <.001 | <.001 | <.001 |
|  | N | 747 | 758 | 758 | 754 | 765 | 730 | 758 | 756 | 752 | 746 | 733 | 758 | 744 | 757 |
| Partner’s bad character | Pearson Correlation | .870^**^ | .637^**^ | .705^**^ | .780^**^ | .621^**^ | 1 | .654^**^ | .681^**^ | .635^**^ | .786^**^ | .720^**^ | .687^**^ | .802^**^ | .693^**^ |
|  | Sig. (2-tailed) | <.001 | <.001 | <.001 | <.001 | <.001 |  | <.001 | <.001 | <.001 | <.001 | <.001 | <.001 | <.001 | <.001 |
|  | N | 725 | 734 | 735 | 730 | 730 | 740 | 734 | 731 | 727 | 722 | 710 | 735 | 725 | 733 |
| In-law and social circle conflicts | Pearson Correlation | .646^**^ | .591^**^ | .551^**^ | .659^**^ | .567^**^ | .654^**^ | 1 | .568^**^ | .570^**^ | .591^**^ | .650^**^ | .556^**^ | .646^**^ | .603^**^ |
|  | Sig. (2-tailed) | <.001 | <.001 | <.001 | <.001 | <.001 | <.001 |  | <.001 | <.001 | <.001 | <.001 | <.001 | <.001 | <.001 |
|  | N | 749 | 762 | 762 | 757 | 758 | 734 | 770 | 759 | 754 | 749 | 736 | 762 | 748 | 761 |
| Bad sex life | Pearson Correlation | .795^**^ | .438^**^ | .561^**^ | .583^**^ | .578^**^ | .681^**^ | .568^**^ | 1 | .592^**^ | .774^**^ | .569^**^ | .733^**^ | .548^**^ | .541^**^ |
|  | Sig. (2-tailed) | <.001 | <.001 | <.001 | <.001 | <.001 | <.001 | <.001 |  | <.001 | <.001 | <.001 | <.001 | <.001 | <.001 |
|  | N | 748 | 761 | 759 | 754 | 756 | 731 | 759 | 767 | 752 | 746 | 735 | 760 | 748 | 760 |
| Partner's neglect of health and appearance | Pearson Correlation | .624^**^ | .591^**^ | .490^**^ | .664^**^ | .639^**^ | .635^**^ | .570^**^ | .592^**^ | 1 | .611^**^ | .651^**^ | .593^**^ | .641^**^ | .705^**^ |
|  | Sig. (2-tailed) | <.001 | <.001 | <.001 | <.001 | <.001 | <.001 | <.001 | <.001 |  | <.001 | <.001 | <.001 | <.001 | <.001 |
|  | N | 743 | 755 | 754 | 749 | 752 | 727 | 754 | 752 | 761 | 744 | 728 | 754 | 741 | 755 |
| Neglect | Pearson Correlation | .853^**^ | .487^**^ | .758^**^ | .708^**^ | .587^**^ | .786^**^ | .591^**^ | .774^**^ | .611^**^ | 1 | .653^**^ | .778^**^ | .569^**^ | .619^**^ |
|  | Sig. (2-tailed) | <.001 | <.001 | <.001 | <.001 | <.001 | <.001 | <.001 | <.001 | <.001 |  | <.001 | <.001 | <.001 | <.001 |
|  | N | 736 | 749 | 747 | 744 | 746 | 722 | 749 | 746 | 744 | 755 | 725 | 749 | 738 | 749 |
| Disagreement over family planning | Pearson Correlation | .656^**^ | .691^**^ | .554^**^ | .762^**^ | .591^**^ | .720^**^ | .650^**^ | .569^**^ | .651^**^ | .653^**^ | 1 | .571^**^ | .730^**^ | .697^**^ |
|  | Sig. (2-tailed) | <.001 | <.001 | <.001 | <.001 | <.001 | <.001 | <.001 | <.001 | <.001 | <.001 |  | <.001 | <.001 | <.001 |
|  | N | 726 | 738 | 736 | 733 | 733 | 710 | 736 | 735 | 728 | 725 | 743 | 736 | 727 | 737 |
| Lack of shared fun and recreation | Pearson Correlation | .785^**^ | .422^**^ | .587^**^ | .578^**^ | .606^**^ | .687^**^ | .556^**^ | .733^**^ | .593^**^ | .778^**^ | .571^**^ | 1 | .535^**^ | .554^**^ |
|  | Sig. (2-tailed) | <.001 | <.001 | <.001 | <.001 | <.001 | <.001 | <.001 | <.001 | <.001 | <.001 | <.001 |  | <.001 | <.001 |
|  | N | 750 | 762 | 761 | 757 | 758 | 735 | 762 | 760 | 754 | 749 | 736 | 770 | 748 | 761 |
| Partner’s jealousy | Pearson Correlation | .703^**^ | .802^**^ | .582^**^ | .737^**^ | .542^**^ | .802^**^ | .646^**^ | .548^**^ | .641^**^ | .569^**^ | .730^**^ | .535^**^ | 1 | .686^**^ |
|  | Sig. (2-tailed) | <.001 | <.001 | <.001 | <.001 | <.001 | <.001 | <.001 | <.001 | <.001 | <.001 | <.001 | <.001 |  | <.001 |
|  | N | 737 | 750 | 748 | 744 | 744 | 725 | 748 | 748 | 741 | 738 | 727 | 748 | 756 | 750 |
| Wasteful with money | Pearson Correlation | .650^**^ | .657^**^ | .583^**^ | .751^**^ | .640^**^ | .693^**^ | .603^**^ | .541^**^ | .705^**^ | .619^**^ | .697^**^ | .554^**^ | .686^**^ | 1 |
|  | Sig. (2-tailed) | <.001 | <.001 | <.001 | <.001 | <.001 | <.001 | <.001 | <.001 | <.001 | <.001 | <.001 | <.001 | <.001 |  |
|  | N | 749 | 763 | 760 | 757 | 757 | 733 | 761 | 760 | 755 | 749 | 737 | 761 | 750 | 768 |
| **. Correlation is significant at the 0.01 level (2-tailed). | | | | | | | | | | | | | | | |
